# A primary gastric synovial sarcoma: a case report and literature review: Erratum

**DOI:** 10.1097/MD.0000000000009531

**Published:** 2017-12-29

In the article, “A primary gastric synovial sarcoma: A case report and literature review”,^[1]^ which appeared in Volume 96, Issue 49 of *Medicine*, the authors would like to make several corrections.

The sentence “When a gastric spindle cell tumor is observed, the possibility of synovial carcinoma, besides common mesenchymal tumor, should also be considered” should appear as “When a gastric spindle cell tumor is observed, the possibility of synovial carcinoma, a rare malignant mesenchymal tumor, should also be considered.”

The sentence “Endoscopic ultrasound showed a 17-mm homogenously hypoechoic mass within the submucosal layer” should appear as “Endoscopic ultrasound showed a 1.7 cm homogenously hypoechoic mass within the submucosal layer.”

The sentence “Endoscopic ultrasound showed a 17-mmhomogenously hypoechoic mass within the submucosal layer (Fig. 2)” should appear as “Endoscopic ultrasound showed a 1.7 cm homogenously hypoechoic mass within the submucosal layer (Fig. 2).”

The sentence “Immunohistochemically, the tumor cells were focally positive for cytokeratin (AE1/AE3), CD99, vimentin, and TEL-1 (Fig. 5)” should appear as “Immunohistochemically, the tumor cells were focally positive for cytokeratin (AE1/AE3), CD99, vimentin, and TLE-1 (Fig. 5).”

The sentence “The cells were negative for chromogranin, synaptophysin, CD119, desmin, S-100, and HMB-45” should appear as “The cells were negative for chromogranin, synaptophysin, CD117, desmin, S-100, and HMB-45.”

The sentence “Immunohistochemistry showed focal positivity for cytokeratin (AE1/AE3) (A), CD99 (B), vimentin (C), and TEL-1 (D)” should appear as “Immunohistochemistry showed focal positivity for cytokeratin (AE1/AE3) (A), CD99 (B), vimentin (C), and TLE-1 (D).”
